# Diversity of KIR/HLA Genotypes and Their Association with Psoriasis Vulgaris in the Western Mexican Population

**DOI:** 10.3390/genes11030338

**Published:** 2020-03-22

**Authors:** Omar Graciano-Machuca, Anabell Alvarado-Navarro, María Guadalupe Ramírez-Dueñas, Delfina Guadalupe Villanueva-Quintero, Erandi Enif Velarde-de la Cruz, Andrea Carolina Machado-Sulbarán, Margarita Montoya-Buelna, Pedro Ernesto Sánchez-Hernández

**Affiliations:** 1 Doctorate in Biomedical Sciences, Department of Physiology, University Center of Health Sciences (CUCS), University of Guadalajara (UDG), Guadalajara, Jal. 44340, Mexicometanarf@gmail.com (E.E.V.-d.l.C.); andrecaroms@gmail.com (A.C.M.-S.); 2 Laboratory of Biological Systems, Department of Health Sciences, University Center of the Valleys (CUVALLES), UDG, Ameca, Jal. 4660, Mexico; 3 Research Center in Immunology and Dermatology, Department of Physiology, CUCS, UDG, Guadalajara, Jal. 44340, Mexico; bell2000_mx@yahoo.com; 4 Laboratory of Immunology, Department of Physiology, CUCS, UDG, Guadalajara, Jal. 44340, Mexico; mgramirezd@gmail.com (M.G.R.-D.);; 5 Jalisco Dermatology Institute “Dr. José Barba Rubio”, Ministry of Health; Zapopan, Jal. 45190, Mexico; 6 Inflammatory Diseases Care Center, Guadalajara, Jal. 44630, Mexico

**Keywords:** psoriasis vulgaris, *KIR*, *HLA*, *KIR3DS1*, *HLA-Bw4*

## Abstract

NK and some T cell functions are regulated by the interaction between KIR and HLA molecules. Several studies have shown an association between activating *KIR* genes and the development of autoimmune diseases, including psoriasis vulgaris (PsV). Our objective was to determine the association between *KIR/HLA* genes and genotypes with PsV in the Western mestizo Mexican population. One hundred subjects diagnosed with PsV (SP) and 108 healthy subjects (HS) were genotyped for 14 *KIR* genes, *HLA-Bw4*, *HLA-C1*, and *HLA-C2* by PCR-single specific primer (SSP). Positive associations of the *KIR3DS1* gene (odds ratio (OR) 1.959, *p* = 0.021), G11 genotype (OR 19.940, *p* = 0.008), and *KIR3DS1/HLA-A^Bw4^* (OR 2.265, *p* = 0.009) were found with susceptibility to PsV. In contrast, the G1 genotype (OR 0.448, *p* = 0.031) and *KIR3DL1/HLA-Bw4^Ile80^* (OR 0.522, *p* = 0.022) were negatively associated with susceptibility to this disease. These results suggest an implication of the *KIR3DS1/HLA-A^Bw4^* genotype in PsV pathology.

## 1. Introduction

Psoriasis is a chronic erythematosquamous dermatosis with a worldwide prevalence in adults between 0.51% and 11.43% [1,2]. Psoriatic lesions are caused by epidermal hyperproliferation with parakeratosis and distributed symmetrically on the skin of the affected individual [3]. There are two major classifications of psoriasis. The first one is based on the age of onset: type I, with an age of onset before 40, and type II after 40 years [4]; the second one is according to the clinical form, of which the most common is psoriasis vulgaris (PsV), with 90% of cases [2]. The disease etiology is multifactorial, where environmental, infectious, immunological, and genetic factors are involved. PsV has a strong genetic background, loci in 424 genes have been reported, mainly HLA-Cw*0602 [5,6].

Characteristic lesions of PsV are plaques, usually affecting the extensor parts of the extremities particularly elbows, knees, scalp, lower lumbosacral region, and genitals [2,6]. Plaque histological changes in PsV are produced by the immune response of the inflammatory infiltrate, composed of NK cells, T cells, and others immune cells [7]. NK cell function is regulated by a complex network of activating and inhibitory signals generated by receptors expressed on their cell membrane, including killer cell immunoglobulin-like receptors (KIR) [8]. Some T cell populations also express KIR receptors and modulate their activation [9,10].

The KIR receptors are encoded by the *KIR* gene family, which is composed of 15 genes and two pseudogenes; it has a size of approximately 150Kb, localized within the leukocyte receptor complex, on chromosome 19q13.4 [8]. Six genes encode for activating receptors (*KIR2DS1-2DS5* and *3DS1*) and seven inhibitors (*KIR2DL1-3*, *2DL5,* and *3DL1-3*), and *KIR2DL4* encodes a receptor that can perform both functions depending on where it is expressed in the cell, as well as two pseudogenes (*KIR2DP1* and *3DP1*) [8,10]. Of these, four are practically present in all individuals and are referred to as framework genes (*KIR3DL3*, *3DP1*, *2DL4*, and *3DL2*) [10,11]. Two types of *KIR* haplotypes are distinguished, designated as A and B. B haplotypes are characterized by containing at least one of the genes: *KIR2DL2*, *2DL5*, *2DS1*-*2DS3*, *2DS5*, *3DS1*; and frequently have more genes than the A haplotypes; whereas the A haplotypes lack the aforementioned genes and tend to be less numerous. The inhibitory KIR receptors have a long cytoplasmic tail, which contains two ITIM motifs, unlike activating KIR receptors that present a short cytoplasmic tail associated with the adapter protein DAP-12, which has two ITAM motifs [8,10].

KIR ligands are HLA class I (HLA-I) molecules. Most KIR/HLA-I interactions have been extensively described [8]. KIR2DS1 and 2DL1 bind to the HLA-C group with residue K80 (HLA-Cw*02, Cw*04, Cw*05, and Cw*06). KIR2DS2, 2DL2, and 2DL3 recognize the HLA-C group with residue N80 (HLA-Cw*01, Cw*03, Cw*07, and Cw*08) [8,10]. KIR3DS1 and 3DL1 recognize the HLA-A and HLA-B expressing the Bw4 epitope; KIR3DL2 recognizes HLA-A3 and A11; KIR2DL4 to HLA-G; KIR2DS4 and 3DL2 to HLA-F. However, for the KIR2DL5, 2DS5, and 3DL3 receptors, the ligands are still unknown [8,10]. 

Studies in Caucasian and Asian populations have suggested positive associations of *KIR2DS1* [12,13,14,15,16,17] and *2DL5* [12,17] genes, as well as the *KIR2DS1*/*HLA*-Cw*0602 genotype with susceptibility to PsV [13,16]. Additionally, in American Caucasian populations, KIR3DL1*Low alleles (alleles expressed at low levels) have been positively associated with susceptibility to PsV [18]; while the KIR3DL1*Null alleles (alleles not expressed at the cell surface) were found to be negatively associated with susceptibility [18,19]. The aim of this study was to elucidate the association between *KIR/HLA-I* genes and genotypes with psoriasis vulgaris in the mestizo population from Western Mexico.

## 2. Materials and Methods

### 2.1. Subjects 

We included 108 healthy subjects (HS) without familiar antecedents of psoriasis and 100 subjects with clinical and histopathological diagnosis of PsV (SP) from the Instituto Dermatológico de Jalisco “Dr. José Barba Rubio”, Jalisco, México, during the period from December 2013 to July 2015. SP were classified according to the age of onset type I (< 40 years) and type II (> 40 years). SP with other autoimmune diseases were excluded. HS were matched with SP according to gender and age. Both groups were mestizos over 18 years from Western Mexico (Aguascalientes, Colima, Guanajuato, Jalisco, Michoacán, Nayarit, and Zacatecas) for at least three generations.

### 2.2. Ethical Approval 

The study was approved by the ethics committee of University Center for Health Sciences, University of Guadalajara, and the Ministry of Health of the State of Jalisco in Mexico (Ethical Approval Code 37/IDJ-JAL/2013); fulfilling the general health law regulations for medical research involving human subjects and the World Medical Association Declaration of Helsinki: ethical principles for medical research involving human subjects [20]. Informed consent was obtained from all participants.

### 2.3. DNA Extraction and KIR/HLA Genotyping

DNA was extracted from a peripheral blood sample using the modified salting-out technique, according to Miller et al. [21]; the sample was resuspended in sterile distilled water and stored at −20 °C until use. DNA samples were genotyped for 14 *KIR* genes (*KIR2DS1-5*, *3DS1*, *2DL1-5*, and *3DL1-3*), two *KIR* pseudogenes (*KIR2DP1* and *3DP1*), *HLABw4* (*HLA-A^Bw4^*, *HLA-Bw4^Ile80^*, and *HLA-Bw4^Thr80^*), and *HLA-C* (*HLA-C1* and *C2*) by polymerase chain reaction-single specific primer (PCR-SSP). Conditions and primers used for *KIR*, *HLA-Bw4*, and *HLA-C* genotyping were according to previously reported methods [22,23,24]. PCR products were visualized by electrophoresis on 3.0% agarose gel Ultrapure TBE buffer 0.5× (Invitrogen™ | Life Technologies, Carlsbad, CA, USA), stained with Sybr Safe solution (Invitrogen™ | Life Technologies, Carlsbad, CA, USA) for 45 min in the dark and photographed with Kodak Molecular Imaging Software V5 (Carestream Health Inc, Rochester, NY, USA).

### 2.4. Statistical Analysis.

The carrier frequencies (CF) of *KIR* genes, *HLA class I* allelic groups, and genotypes (AA and Bx gene-content, *KIR* combined, and *KIR/HLA* composed) were obtained by direct counting. Gene frequencies (GF) of *KIR* genes and *HLA* allelic groups were determined by Bernstein’s formula: GF=1−√(1−F). (using CF) [25]. For the *KIR* genotype profile, Hardy–Weinberg equilibrium was calculated in both groups. The comparisons of the gene and genotype frequencies were performed with the X^2^; odds ratios (OR) with 95% confidence interval (95% CI) were estimated for susceptibility association using GraphPad Prism Software Version 8.02. *p* < 0.05 values were considered statistically significant and adjusted with Holm–Bonferroni correction for multiple comparisons. PS Power and the Sample Size Calculation Version 3.1.6 program were used to calculate the statistical power of this study, which ranged between 90 and 100%, except for three analyses, which were specified in the Results Section. Linkage disequilibrium (LD) values for *KIR* genes were assessed using the Wn statistic (Cramer’s V statistic):

*Wn*^*=((ad-bc))⁄(√((a+b)(c+d)(a+c)(b+d))).

## 3. Results

### 3.1. Clinical and Demographic Characteristics

One hundred eight HS were included in this study and 100 SP. The mean age for HS was 48.5 ± 12.5 years (range 18–79); the gender proportion was 1:1 (54 females and 54 males). In SP, the mean age was 47.7 ± 14.7 years (range 18–76); and the gender proportion was 1:1 (50 females and 50 males). Thirty-one SP reported family background affected by psoriasis (31%). Type I psoriasis (onset before age 40) was reported in 62 SP; whereas type II psoriasis (onset after 40 years) in 38 SP. Type I SP showed a higher frequency of family background than type II SP (37% vs 21%), without a significant difference (*p* = 0.096).

### 3.2. Frequencies of KIR Genes and HLA Class I Alleles

*KIR* and *HLA class I* gene frequencies were compared between HS and SP (Table 1). A significant increase in the carrier frequency of *KIR3DS1* was found in SP compared to HS (OR 1.959, 95% CI 1.102–3.481, *p* = 0.021) before Holm–Bonferroni correction. Framework genes (*KIR2DL4*, *3DL2*, *3DL3*, and *3DP1*) were identified in almost all studied participants. The other *KIR* genes (*KIR2DL1*, *2DL2*, *2DL3*, *3DL1*, *2DS1*, *2DS2*, *2DS3*, *2DS4*, *2DS5*, *2DP1*, and *2DL5*) showed similar carrier frequencies, without significant differences between SP and HS.

The *HLA-A^Bw4^* allelic group was significantly higher in SP than in HS before Holm–Bonferroni correction, but with low statistical power (OR 1.769, CI 95% 1.016-3.081, *p* = 0.044; statistical power = 69.95%). The *HLA-Bw4^Ile80^* allelic group showed a lower frequency in SP than in HS (OR 0.517, CI 95% 0.293-0.911, *p* = 0.023) before Holm–Bonferroni correction. The frequencies of *HLA-C1* and -*C2* groups were similar in SP and HS. However, the statistical significances of *KIR3DS1*, *HLA-A^Bw4^*, and *HLA-Bw4^Ile80^* were lost after Holm–Bonferroni correction for multiple comparisons (*p’* = 0.336, *p’* = 0.264, and *p’* = 0.138, respectively).

### 3.3. KIR Genotypes Profiles

The *KIR* genotype profiles are shown in Table 2; sixty different *KIR* genotypes were found: 21 in SP, 19 in HS, and 20 shared in both groups. A significant decrease of genotype 1 (ID 1, according to the Allele Frequency Net Database, consulted in 2020 [26]) in SP compared to HS (OR 0.448, 95% CI 0.217–0.928, *p* = 0.031) was found. On the other hand, genotype 11 (ID 14, according to the Allele Frequency Net Database, consulted in 2020 [26]) showed a statistically significant increase in SP compared to HS (OR 19.940, 95% CI 1.135-350.400, *p* = 0.008).

*KIR* genotypes were classified as AA and Bx according to the *KIR* gene content. Although the AA genotype frequency was higher in HS than in SP (25% *vs.* 15%, respectively), and consequently Bx genotypes in the SP compared with HS (85% vs. 75%, respectively), there were no significant differences (*p* = 0.075).

### 3.4. KIR Combined Genotypes

Due to the positive association of the *KIR3DS1* gene with the susceptibility to psoriasis, *KIR3DS1* combined genotypes analysis were performed. The main *KIR* combined genotype frequencies are shown in Table 3. *KIR3DS1-/2DS2-* and *KIR3DS1-/2DL5-* genotypes were significantly lower in SP than in HS (OR 0.486, 95% CI 0.250–0.946, *p* = 0.034 and OR 0.470, 95% CI 0.253–0.875, *p* = 0.017; respectively); however, the *KIR3DS1-/2DL5*- genotype showed low statistical power (58.91%). On the other hand, the *KIR3DS1+/2DL5*- and *KIR3DS1+/2DS3*- genotypes showed increased frequency in SP compared to HS (OR 3.482, 95% CI 1.314–9.229, *p* = 0.012 and OR 1.769, 95% CI 1.102–3.077, *p* = 0.043; respectively). However, after Holm–Bonferroni correction, differences in the combined genotype lost significance. Linkage disequilibrium (LD) of *KIR3DS1* with *2DS2*, *2DS3*, *2DL2*, and *2DL5* was calculated in HS (Wn^2DS2^ = 0.229, Wn^2DS3^ = 0.236, Wn^2DL2^ = 0.215, and Wn^2DL 5^ = 0.701; *p* = 0.0001) and SP (Wn^2DS2^ = 0.175, Wn^2DS3^ = 0.233, Wn^2DL2^ = 0.211, and Wn^2DL5^ = 0.453; *p* = 0.0001).

### 3.5. KIR/HLA Composed Genotype Frequencies

The *KIR/HLA* genotype frequencies in both groups were analyzed and are shown in Table 3. The *KIR3DS1/HLA-A^Bw4^* activating genotype showed a positive association with susceptibility to PsV (OR 2.265, 95% CI 1.228–4.180, *p* = 0.009); while the inhibitory genotype *KIR3DL1/HLA-Bw4^Ile80^* was negatively associated with susceptibility to PsV (OR 0.522, 95% CI 0.299–0.910, *p* = 0.022), but with low statistical power (59.24%). The *KIR/HLA-C* genotypes presented an equal distribution between groups. The significance of the composed genotypes was lost after Holm–Bonferroni correction.

### 3.6. Association of the iKIR and aKIR Number with Susceptibility to PsV

**Figure 1:**
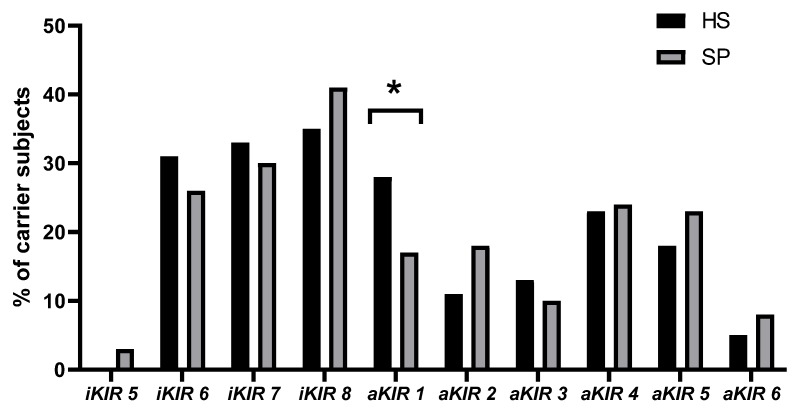
Distribution of inhibitory and activating KIR genes in healthy subjects (HS) and subjects with psoriasis vulgaris (SP). * *p* = 0.047 based on X^2^ and *p*’ = 0.282, based on Holm–Bonferroni correction for multiple comparisons.

Genotypes were evaluated according to the number of inhibitory and activating *KIR* genes and are shown in Figure 1. Genotypes with a single activator gene were increased significantly in HS compared to SP (OR 0.509, 95% CI 0.261–0.922, *p* = 0.047), but the significance of this difference disappeared after Holm–Bonferroni correction (*p’* = 0.282).

## 4. Discussion

Here, we report for the first time the association of *KIR/HLA* genes and genotypes with PsV susceptibility in the Western Mexican mestizo population. Our *KIR* gene frequency data in HS showed similarity with previous reports from the same region [27,28]. The comparison with SP suggested that the *KIR3DS1* gene was a susceptibility factor for PsV in our studied population. Similarly, a study conducted in the European population in 2012 by Chen et al. found an association between *KIR3DS1* gene presence and PsV susceptibility (SP = 46.6% vs. HS = 32.6%, *p* < 0.001) [29]. The *KIR3DS1* gene has also been associated with susceptibility to psoriatic arthritis, a seronegative spondyloarthropathy related to psoriasis disease [30]. In contrast to our results, studies in Japanese, Polish, American, Swedish, and Brazilian populations have shown a positive association mainly with the *KIR2DS1* gene [12,13,14,15,16]. However, we did not find an association with *KIR2DS1*; a possible explanation for this could be related to the HLA-Cw*0602 allele, as described by Dunphy et al. who suggested that the association between the *KIR2DS1* gene and psoriasis could be due to the presence of HLA-Cw*0602 and not to *KIR2DS1* [31]. However, it is important to note that we did not typify this *HLA-C* allele.

Concerning *HLA* frequencies, our results showed that *HLA-A^Bw4^* and *HLA-Bw4^Ile80^* allelic frequencies were higher and lower respectively in SP than in HS. The *HLA-C* frequencies were similarly distributed between HS and SP. Contrary to our results, Ahn et al. reported no association of the *HLA-Bw4* allelic group with psoriasis [18]; while Berinstein et al. found an increased *HLA-Bw4^Ile80^* frequency in PS compared to HS [19]; both studies were performed in American Caucasian populations. However, it would be interesting to evaluate the association of the specific *HLA class I* alleles to psoriasis in our population, such as HLA-Cw*0602, which has been the main susceptibility factor for this pathology [32].

The current study revealed 60 *KIR* genotype profiles; three of them (G58–G60) have not been previously reported in the Allele Frequency Net Database [26]. The genotype identified as G1, an AA genotype, was a protective factor in our population. This genotype is composed of six genes encoding inhibitory receptors and only a gene encoding an activating receptor, which could be limiting the inflammatory process. In the same population, Ramírez-De Los Santos et al. found this genotype (G1) as a protective factor against rheumatoid arthritis [27]. On the other hand, we found that the G14 genotype, a Bx genotype, which contains the *KIR3DS1* gene, possibly was a susceptibility factor for PsV in our population due to its increased frequency in SP. When *KIR* genotypes profiles were classified according to the gene content, we observed that Bx genotypes were more frequent in our study population. The Bx and AA genotype frequencies found in this study agreed with previous reports from our population [27,28]. Studies in autoimmune diseases, such as type 1 diabetes, systemic sclerosis, rheumatoid vasculitis, and rheumatoid arthritis, have shown an increased frequency of Bx genotypes in patients compared to the control group, which could be since that Bx genotypes, compared to AA genotypes, usually contain a greater number of genes that encode activating receptors, including KIR3DS1 [27,33,34].

When we analyzed the genotype formed by the receptor and its ligand genes (composed genotype), we observed an increased frequency of *KIR3DS1/HLA-A^Bw4^* in SP compared with HS, while the *KIR3DL1/HLA-Bw4^Ile80^* genotype frequency was increased in HS. In this sense, several studies reported that the *KIR/HLA class I* genotypes could be associated with PsV development. Ahn et al. investigated the association of *KIR3DL1* alleles and *HLA*-*Bw4*, and they found a positive association of the KIR3DL1*Low/HLA-Bw4 composed genotype with susceptibility to PS [18]; however, unlike our study, they performed a *KIR3DL1* allelic typing and determined the presence of the HLA-Bw4 epitope. Recently, Berinstein et al. reported a negative association of KIR3DL1*Null alleles with psoriasis susceptibility [19]; however, in this study, *KIR3DL1* alleles/*HLA-Bw4* composed genotypes were not analyzed. 

According to our results, we could hypothesize that the receptors KIR3DS1 and KIR3DL1 could modulate the development of psoriasis through binding to their ligands in two aspects. The first of these could occur during the NK cell licensing process that involves signaling generated through inhibitory receptors. Alter et al. suggested that high levels of KIR3DL1 expression and binding to HLA-Bw4^Ile80^ molecules in the process of licensing could generate NK cells with a more efficient cytotoxic function, as opposed to when there is a low expression of this receptor [35]. In contrast, recognition by activating receptors such as KIR2DS1 has been associated with decreased cytotoxic capacity against the target cell [36]. The second aspect would occur during the effector phase; KIR3DS1 could activate NK cells and some T cells populations through the accessory protein DAP-12 [37]. HLA-Bw4 and KIR3DS1 interaction could stimulate the IFN-γ and other cytokines overproduction, and this, in turn, could induce keratinocyte proliferation and secretion of adhesion molecules, chemokines, and proinflammatory cytokines related to the lesion initiation and feedback [38,39]. Nevertheless, the inflammatory process could be decreased by HLA-Bw4^Ile80^ recognition through KIR3DL1. Inhibitory KIR block downstream signals that are generated by activating receptors [39].

## 5. Conclusions

This study reported for the first time an association of *KIR* genes and genotypes with PsV susceptibility in the Western Mexican mestizo population. We found the *KIR3DS1* gene and *KIR3DS1/HLA-A^Bw4^* genotype as susceptibility factors for PsV, whereas the *KIR3DL1/HLA-Bw4^Ile80^* genotype was found as a protective factor. This may suggest that NK and T cells could modulate the inflammatory process in PsV through the binding of KIR3DL1 and 3DS1 receptors to HLA-Bw4 alleles. However, it is necessary to perform functional expression studies to elucidate the role of KIR receptors in the PsV pathology.

## Figures and Tables

**Table 1:** Distribution of *KIR* and *HLA* genes in healthy subjects and subjects with psoriasis vulgaris.

Gene	HS (*n* = 108)			SP (*n* = 100)						
	*nCF*	%CF	GF	*nCF*	%CF	GF	*p*	*OR*	95% CI	*p’*
***KIR***										
*2DL1*	108	100.0	1.00	99	99.0	0.90	NS			
*2DL2*	59	54.6	0.33	61	61.0	0.38	NS			
*2DL3*	107	99.1	0.90	96	96.0	0.80	NS			
*3DL1*	101	93.5	0.75	92	92.0	0.72	NS			
*3DL2*	106	98.1	0.86	99	99.0	0.90	NS			
*2DS1*	52	48.1	0.28	52	52.0	0.31	NS			
*2DS2*	51	47.2	0.27	55	55.0	0.33	NS			
*2DS3*	24	22.2	0.12	21	21.0	0.11	NS			
*2DS4*	101	93.5	0.75	92	92.0	0.72	NS			
*2DS5*	47	43.5	0.25	47	47.0	0.27	NS			
***3DS1***	**60**	**55.6**	0.33	**71**	**71.0**	0.46	**0.021**	**1.959**	**1.102–3.481**	**0.336**
*2DP1*	107	99.1	0.90	99	99.0	0.90	NS			
*3DL3*	108	100.0	1.00	100	100.0	1.00	NS			
*2DL4*	108	100.0	1.00	100	100.0	1.00	NS			
*2DL5*	63	58.3	0.35	62	62.0	0.38	NS			
*3DP1*	108	100.0	1.00	100	100.0	1.00	NS			
***HLA***										
*C1*	107	99.1	0.90	98	98.0	0.86	NS			
*C2*	100	92.6	0.73	90	90.9	0.70	NS			
*A (Bw4)*	**39**	36.1	0.20	50	**50.0**	0.29	0.044	1.769	**1.016–3.081**	**0.264**
*Bw4Ile80*	**75**	69.4	0.45	54	**54.0**	0.32	0.023	0.517	**0.293–0.911**	**0.138**
*Bw4Thr80*	62	57.4	0.35	43	43.0	0.25	NS			
Bw4*	98	90.7	0.70	90	90.0	0.68	NS			

HS: Healthy Subjects; SP: Subjects with Psoriasis vulgaris; KIR: Killer Immunoglobulin-like Receptor; HLA: Human Leukocyte Antigen; CF: Carrier Frequency; GF: Gene Frequency; OR: Odds Ratio; CI: Confidence Interval; NS: Not Significant; *p* based on X^2^ test and adjusted *p*’ based on Holm-Bonferroni correction for multiple comparisons. Significant differences between groups are in bold. * At least one positive HLA-Bw4 reaction.

**Table 2:** *KIR* genotype profile of healthy subjects and subjects with psoriasis vulgaris.

GENOTYPE			*KIR GENES*																HS	SP
G	ID	AA and Bx gene-content genotypes	*3DL1*	*2DL1*	*2DL3*	*2DS4*	*2DL2*	*2DL5*	*3DS1*	*2DS1*	*2DS2*	*2DS3*	*2DS5*	*2DL4*	*3DL2*	*3DL3*	*2DP1*	*3PD1*	*n* = 108	*n* = 100
G1*	**1**	**AA**																	**27**	**13**
G2	2	Bx																	12	9
G3	3	Bx																	13	14
G4	4	Bx																	6	4
G5	5	Bx																	4	2
G6	6	Bx																	2	5
G7	7	Bx																	3	4
G8	8	Bx																	1	0
G9	9	Bx																	1	1
G10	13	Bx																	2	2
G11**	**14**	**Bx**																	**0**	**8**
G12	15	Bx																	1	1
G13	18	Bx																	3	0
G14	19	Bx																	2	0
G15	20	Bx																	0	1
G16	22	Bx																	1	1
G17	27	Bx																	1	0
G18	28	Bx																	1	0
G19	29	Bx																	0	1
G20	31	Bx																	3	1
G21	33	Bx																	1	0
G22	38	Bx																	1	1
G23	44	Bx																	2	0
G24	49	Bx																	0	1
G25	56	Bx																	1	1
G26	58	Bx																	0	1
G27	69	Bx																	2	1
G28	70	Bx																	1	2
G29	75	Bx																	0	1
G30	80	Bx																	0	3
G31	86	Bx																	0	1
G32	87	Bx																	1	1
G33	93	Bx																	1	0
G34	94	Bx																	0	1
G35	136	Bx																	0	1
G36	137	Bx																	0	1
G37	167	Bx																	1	0
G38	180	AA																	0	1
G39	184	Bx																	1	0
G40	188	Bx																	1	3
G41	191	Bx																	1	0
G42	200	Bx																	0	1
G43	240	Bx																	0	1
G44	260	Bx																	1	0
G45	266	Bx																	1	0
G46	299	Bx																	1	0
G47	319	Bx																	0	1
G48	328	Bx																	2	1
G49	336	Bx																	0	1
G50	381	Bx																	0	1
G51	383	Bx																	0	2
G52	587	Bx																	1	2
G53	650	AA																	0	1
G54	654	Bx																	1	0
G55	657	Bx																	1	0
G56	680	Bx																	1	0
G57	720	Bx																	0	1
G58	NR	Bx																	1	0
G59	NR	Bx																	1	0
G60	NR	Bx																	0	1

HS: healthy subjects; SP: subjects with psoriasis vulgaris; G: genotype identified assigned in this study; ID: ID González-Galarza (January 2020); NR: not reported, ID; black box = gene detected; white box = gene absent. Significant differences between both groups are in bold. * *p* = 0.031, OR = 0.448, 95% CI: 0.217 to 0.928; *p*’ = 1.000, ** *p* = 0.008, OR = 19.94, 95% CI: 1.135 to 350.400; *p* ’= 0.480.

**Table 3:** Distributions of *KIR* combined and *KIR/HLA* composed genotypes in healthy subjects and subjects with psoriasis vulgaris.

Genotypes	HS (*n* = 108)		SP (*n* = 100)					
	*n*	%	*n*	%	*p*	*OR*	95% CI	*p’*
**Combined**								
*KIR3DS1+/2DS2+*	34	31.5	43	43.0	NS			
*KIR3DS1+/2DS2-*	25	23.1	28	28.0	NS			
*KIR3DS1-/2DS2+*	17	15.7	12	12.0	NS			
***KIR3DS1-/2DS2-***	**32**	**29.6**	**17**	**17.0**	**0.034**	**0.486**	**0.250–0.946**	0.544
*KIR3DS1+/2DL2+*	38	35.2	48	48.0	NS			
*KIR3DS1+/2DL2-*	21	19.4	23	23.0	NS			
*KIR3DS1-/2DL2+*	21	19.4	13	13.0	NS			
*KIR3DS1-/2DL2-*	28	25.9	16	16.0	NS			
*KIR3DS1+/2DL5+*	53	49.1	54	54.0	NS			
***KIR3DS1+/2DL5-***	**6**	**5.6**	**17**	**17.0**	**0.012**	**3.482**	**1.314–9.229**	0.192
*KIR3DS1-/2DL5+*	10	9.3	8	8.0	NS			
***KIR3DS1-/2DL5-***	**39**	**36.1**	**21**	**21.0**	**0.017**	**0.470**	**0.253–0.875**	0.272
*KIR3DS1+/2DS3+*	19	17.6	20	20.0	NS			
***KIR3DS1+/2DS3-***	**40**	**37.0**	**51**	**51.0**	**0.043**	**1.769**	**1.102–3.077**	0.688
*KIR3DS1-/2DS3+*	6	5.6	2	2.0	NS			
*KIR3DS1-/2DS3-*	43	39.8	27	27.0	NS			
**Composed**								
*KIR3DL1/HLA-A^Bw4^*	35	32	45	45.0	NS			
***KIR3DL1/HLA-Bw4^Ile80^***	**70**	**65**	**49**	**49.0**	**0.022**	**0.522**	**0.299–0.910**	0.286
*KIR3DL1/HLA-Bw4^Thr80^*	58	54	41	41.0	NS			
KIR3DL1/HLA-Bw4*	91	84	83	83.0	NS			
***KIR3DS1/HLA-A^Bw4^***	**23**	**21**	**38**	**38**	**0.009**	**2.265**	**1.228–4.180**	0.117
*KIR3DS1/HLA-Bw4^Ile80^*	44	41	40	40	NS			
*KIR3DS1/HLA-Bw4^Thr80^*	36	33	26	26	NS			
KIR3DS1/HLA-Bw4*	56	52	64	64	NS			
*KIR2DL1/HLA-C2*	104	96	90	90	NS			
*KIR2DS1/HLA-C2*	48	44	46	46	NS			
*KIR2DL2/HLA-C1*	59	55	61	61	NS			
*KIR2DL3/HLA-C1*	106	98	94	94	NS			
*KIR2DS2/HLA-C1*	51	47	53	53	NS			

HS: Healthy Subjects; SP: Subjects with Psoriasis vulgaris; KIR: Killer Immunoglobulin-like Receptor; HLA: Human Leukocyte Antigen; *p* based on X^2^ test and adjusted *p*’ based on Holm-Bonferroni correction for multiple comparisons; NS: Not Significant; OR = Odds Ratio; CI: Confidence Interval; Significant differences between groups are in bold. * At least one positive HLA-Bw4 reaction.
